# Acetylation Modification During Autophagy and Vascular Aging

**DOI:** 10.3389/fphys.2021.598267

**Published:** 2021-03-22

**Authors:** Jiaxing Sun, Shi Tai, Liang Tang, Hui Yang, Mingxian Chen, Yichao Xiao, Xuping Li, Zhaowei Zhu, Shenghua Zhou

**Affiliations:** Department of Cardiology, The Second Xiangya Hospital of Central South University, Changsha, China

**Keywords:** autophagy, acetylation, FOXO1, SIRT1, vascular aging

## Abstract

Vascular aging plays a pivotal role in the morbidity and mortality of elderly people. Decrease in autophagy leads to acceleration of vascular aging, while increase in autophagy leads to deceleration of vascular aging. And emerging evidence indicates that acetylation plays an important role in autophagy regulation; therefore, recent research has focused on an in-depth analysis of the mechanisms underlying this regulation. In this review, current knowledge on the role of acetylation of autophagy-related proteins and the mechanisms by which acetylation including non-autophagy-related acetylation and autophagy related acetylation regulate vascular aging have been discussed. We conclude that the occurrence of acetylation modification during autophagy is a fundamental mechanism underlying autophagy regulation and provides promising targets to retard vascular aging.

## Introduction

Aging is a process of functional decline of life and is associated with an increase in age-related diseases (Lopez-Otin et al., 2013). With increase in the life expectancy of humans, the incidence of age-related pathologies is also increasing and exceeds the sustainability of economy and society. As a matter of result, it is urgent to find effective interventions to prevent the deterioration of age-associated conditions. Therefore, research is being performed on identifying potential mechanisms underlying aging and effective ways to retard aging.

Post-translational modifications (PTMs) play a pivotal role in determining the structure, destination, activity, and function of proteins. PTMs includes phosphorylation, glycosylation, ubiquitination, lipidation, methylation, and acetylation (Wani et al., 2015). Various factors, for example, nutrient availability, and proper organellar function determine the kind of PTMs that a protein undergoes, and different PTMs are tightly regulated (Wani et al., 2015; Xie et al., 2015). An increasing number of recent studies have indicated that acetylation, a kind of PTMs, is an important regulator of autophagy. Autophagy is a predominantly cytoprotective process, and the autophagic rate decreases with increase in age (Rubinsztein et al., 2011). Emerging evidence shows that autophagy plays a prominent role in life span determination and age-related conditions, including those involving the respiratory system (Kuwano et al., 2016) or the immune system (Zhang et al., 2016) and conditions such as cardiovascular diseases (Stern et al., 2003; Shirakabe et al., 2016; Humphrey and Milewicz, 2017; Ren and Zhang, 2018), neurodegeneration (Fivenson et al., 2017; Plaza-Zabala et al., 2017), and bone aging (Ma et al., 2018). Autophagy is a multistep process involving autophagosome formation and content degradation by lysosomes. Researchers are focusing on whether regulating the acetylation of autophagy-related proteins can enhance autophagy, thereby partly reverting aging and age-related conditions including vascular aging.

Bánréti et al. (2013) has summarize how previously identified histone acetylases (HATs) and deacetylases (HDACs) modify key autophagic effector proteins, and discussed how they play a role in neurodegenerative diseases and cancer. In the current article, we have added the research of acetylation modification during the multistep process of autophagy in the recent years, discussed the existed researches about acetylation in vascular aging, and suggested regulating the acetylation of autophagy may be a potential way to retard vascular aging.

## Autophagy and Vascular Aging: An Overview

Autophagy is a cytoprotective process and includes three different types, which are macro-autophagy, micro-autophagy, and chaperone-mediated autophagy (CMA). Macro-autophagy is the principal and most commonly studied type of autophagy, and is commonly referred as autophagy (Ktistakis and Tooze, 2016). Classical autophagy involves five steps: initiation, nucleation, vesicle elongation, autophagosome maturation and lysosome infusion, degradation, the detailed processes have been shown in these literatures (Khan et al., 2016; Leidal et al., 2018; Dossou and Basu, 2019). In the recent years, the effects of autophagy in vascular aging have been extensively studied. A growing number of studies have provided evidence that autophagy is a fundamental process to ensure vascular health during aging, and compromised autophagic functions may be important in the development of aging (Jiang, 2016; Abdellatif et al., 2018). Thus, regulating autophagy in vessels is a promising way to prevent vascular aging and age-related vascular diseases.

## Acetylation Modification During the Different Processes of Autophagy

Acetyl-CoA (Ac-CoA), acetyltransferases, deacetyl transferases, and targeted protein sites are the three main elements of acetylation modification. Ac-CoA is the only source of acetyl (Pietrocola et al., 2015), and acetyltransferases and deacetyl transferases transfer or remove the acetyl to or from the targeted proteins. HATs and HDACs comprise acetyl–deacetyl transferase pairs (Peserico and Simone, 2011). Acetylation was first detected nearly 50 years ago, histones containing lysine were the first and extensively studied target proteins of acetylation modification, while some non-histones including transcription factors and cytoplasmic proteins were also targeted proteins (N-termini of target proteins),which regulate energy metabolism, endocytosis, and cytoskeleton (Deribe et al., 2010). Bánréti et al. (2013) reviewed different classes of HATs and HDACs. HATs fall into three major classes: lysine acetyltransferase (KAT)2/general control non-derepressible 5 (GCN5)-related N-acetyltransferases (GNAT family), E1A-binding protein p300 (EP300; CREBBP family), and the MYST family. Deacetylases are also divided into several classes: class I, IIa, IIb, and zinc-dependent class IV enzymes; class III family, which uses NAD^+^ to complete deacetylation reactions. Class I consists of HDAC1-3 and HDAC8; class IIa includes HDAC4, HDAC5, HDAC7, and HDAC9; class IIb includes HDAC6 and HDAC10; class IV includes HDAC11; and the class III family comprises sirtuins (SIRT), including SIRT1–7 (Sadoul et al., 2011; Narita et al., 2019).

### Acetylation and Autophagy Initiation

In mammalian cells, autophagy is induced by unc-51-like kinase 1 (ULK1) [Autophagy-related gene 1 (ATG1), yeast homolog)], which interacts with ATG13, FAK family kinase-interacting protein of 200 kDa [FIP200 (ATG17, yeast homolog)], and ATG101 (no known yeast homolog) (Hosokawa et al., 2009; Zachari and Ganley, 2017; Mercer et al., 2018). ULK1 is mainly regulated by mammalian target of rapamycin (mTOR) (Dossou and Basu, 2019). Under growth factor deprivation, human immunodeficiency virus (HIV) Tat-interactive protein 60 kDa (TIP60), an acetyltransferase can regulate autophagy by activating ULK1 (Lin et al., 2012a,b). Under endoplasmic reticulum (ER) stress, autophagy can also be modulated through the same way (Nie et al., 2016). Under both growth factor deprivation and ER stress, the glycogen synthase kinase-3β (GSK3β)/TIP60/ULK1 pathway is engaged to increase autophagy. In these studies, it was found that GSK3β first phosphorylated TIP60-Ser86 and activated TIP60. Then, the activated TIP60 directly acetylated ULK1 and activated the protein kinase. The acetylation sites on ULK1 are located at K162 and K606. Researchers found that in ULK1^–/–^ mouse embryonic fibroblasts, the acetylation-defective mutant ULK1 showed decreased kinase activity and failed to rescue autophagy, which further proved that acetylation is vital to ULK1 activation (Lin et al., 2012b). These studies revealed that induction of autophagy on ULK1 activation integrates phosphorylation and acetylation modification.

### Acetylation During Nucleation in Autophagy

On specific membranes, phosphoinositide-3-kinase class 3 (PIK3C3) or vacuolar protein sorting 34 (VPS34) combines with its regulatory proteins to form the PIK3C3/VPS34-Beclin1 (BECN1)-PIK3R4/VPS15/p150 core-based complex. This process is essential for PIK3C3/VPS34 to exert its lipid kinase activity in cells, thus promoting the nucleation of autophagy. Through interacting with PIK3C3/VPS34 or recruiting it to the specific membranes, these regulatory proteins affect nucleation of autophagy. Both *in vitro* and *in vivo* trials, evidence shows that EP300-dependent acetylation turns PIK3C3/VPS34 off, while deacetylation turns PIK3C3/VPS34 on, wherein deacetylation site K771 is required for its full activation. The PIK3C3/VPS34 activation mechanism referred above happens not only in starvation-induced autophagy but also in the process of autophagy that does not involve Adenosine 5′-monophosphate-activated protein kinase (AMPK), mTORC1, or ULK1 (Su et al., 2017; Kary, 2018; Su and Liu, 2018).

A study found that the phosphorylation of BECN1 at S409 is required for the subsequent BECN1 acetylation, which is mediated by p300 at lysine 430, and SIRT1 can reverse acetylation of BECN1 at lysine 437 (Sun et al., 2015). BECN1 is also regulated via several different types of ubiquitination (Boutouja et al., 2017). Acetylated inducible heat shock protein (HSP)70 increases under autophagy-inducing stress, and HSP70 binds to the BECN1-VPS34 complex. The mechanism is that acetylated HSP70 recruits KRAB-ZFP-associated protein 1 (KAP1), an E3 ligase, for SUMOylation, thereby inducing Lys840 SUMOylation and increasing the activity of VPS34 bound to BECN1 (Yang et al., 2013). TRIM (Tripartite motif), an E3 ligase protein family, enhances the binding of BECN1 with ULK1 and promotes autophagy activities via BECN1 ubiquitination; The Lys-372 residue of TRIM50, critical for its acetylation, is also necessary for its E3 ligase activity controlling BECN1 ubiquitination; this reveals acetylation–ubiquitination-dependent control of autophagy modulation (Fusco et al., 2018). Similar to the phosphorylation–acetylation cascade in ULK1 acetylation, the acetylation–ubiquitination cascade is vital for the regulation of autophagy by BECN1. In summary, BECN1 acetylation inhibits autophagosome maturation, leading to impairment of autophagic flux (Esteves et al., 2019). Studies targeting BECN1 found that supplementation of omega-3 polyunsaturated fatty acid induces the autophagy pathway through upregulation of SIRT1-mediated deacetylation of BECN1, thus attenuates neuronal apoptosis in traumatic brain injury (Chen et al., 2018). In colorectal cancer cells, aspirin induces autophagosome formation, while aspirin-mediated BECN1 acetylation blocks autophagic degradation (Sun et al., 2017). The result indicates that if deacetylation in autophagy is enhanced, aspirin-induced autophagy will also be enhanced.

### Acetylation Modification During Autophagy Vesicle Elongation

In yeast, researchers found that ATG3 is deacetylated by HDAC Rpd3, which controls its interaction with ATG8 and regulate autophagy by affecting the dynamics, flux, and duration of autophagy shortly after induction of starvation; KAT5/TIP60 (mammalian homolog of Esa1) can also regulate autophagy by ATG3 acetylation (Yi and Yu, 2012). Under the condition of nutrient deprivation, nuclear microtubule-associated proteins light chain 3 (LC3); LC3 deacetylation by SIRT1 deepens its communication with the nuclear factor TP53INP2/DOR; As a result, It causes the distribution of nuclear LC3 to the cytoplasm, LC3 then interacts with ATG7, leading to LC3 lipidation and autophagosome biogenesis (Liu and Klionsky, 2015). This process referred above is highly regulated by the nuclear deacetylase SIRT1 (deacetylation at K49 and K51), which helps LC3 translocated from nuclear to cytoplasm (Huang et al., 2015). In HepG2 cells, enhanced binding of SIRT1-LC3 reduces the endogenous LC3 acetylation and SIRT1 inactivation inhibits autophagy (Li X. et al., 2016). Increased SIRT1 expression decreases ATG5 acetylation on lysine residues and increases autophagy (Jiang et al., 2016). While preventing p300 autoinhibition and promoting LC3 acetylation impedes LC3 lipidation (Wan et al., 2017). HLA-B–associated transcript 3 (BAT3) increases p53 acetylation and the expression of its target gene; while it limits p300-dependent acetylation of ATG7; Thus, we conclude that BAT3 highly regulate autophagy by modulating the localization of intracellular p300, thereby affecting the p300 targeted on its substrates, p53 and ATG7 (Sebti et al., 2014). In summary, SIRT1 and p300 comprise a key pair of regulators in acetylation modification during autophagy vesicle elongation. During this stage, protein deacetylation activates autophagy, whereas acetylation inhibits autophagy.

### Acetylation Modification During Autophagosome Maturation

Acetylation occurring upstream of autophagy also affects the later stages of autophagy. BECN1 acetylation negatively regulates autophagosome maturation by inhibiting Rubicon recruitment (Matsunaga et al., 2009; Zhong et al., 2009). Although lysosomal biogenesis seems to be triggered as a compensatory response when BECN1 acetylation impairs autophagic flux, autophagosome fusion with lysosomes is compromised, contributing to Alzheimer’s disease (AD) neurodegeneration (Esteves et al., 2019). The GSK3-TIP60 pathway (Lin et al., 2012a,b; Nie et al., 2016), involved in an early stage of autophagy, also regulates autophagosome maturation mediated by autophagy regulator–RUBCNL/pacer acetylation (Cheng and Sun, 2019; Cheng et al., 2019), which is the opposite of the regulating results for RUBCN/Rubicon. RUBCNL and RUBCN compose a dual molecular switch model controlling autophagosome maturation (Cheng and Sun, 2019). Furthermore, LC3B-II deacetylation, which is partly mediated by HDAC6, increases the degradation of p62/SQSTM1 and is involved in autophagic degradation during serum starvation (Liu et al., 2013).

Researches have proven that HDAC6 deacetylase domain is also required for aggresomal formation, autophagosome–lysosome fusion, and autophagic turnover (Kawaguchi et al., 2003; Iwata et al., 2005; Pandey et al., 2007). And researchers also found that SIRT1 may compensate for the function of HDAC6. Cortactin deacetylation mediated by cytosolic HDAC6 is required for autophagosome–lysosome fusion (Lee et al., 2010). SIRT1 is predominantly located in the cytosol (Li et al., 2008), where SIRT1 interacts with cortactin and deacetylates it. Both HDAC6 and SIRT1 have been found that they can bind and deacetylate cortactin independently. However, depending on specific cell types or tissues, HDAC6 and SIRT1 can also work cooperatively or competitively (Zhang et al., 2009). For example, in HeLa cells, either HDAC6 or SIRT1 can bind and deacetylate cortactin independently, and they can also work cooperatively toward cortactin (Zhang et al., 2007). Either SIRT1 or HDAC6 binds to the same domain of cortactin, thus, researchers speculate that SIRT1 and HDAC6 may compete for the domain to bind cortactin in OV2008 cells (Zhang et al., 2009). Moreover, ATP13A2 can recruit HDAC6 to lysosomes to deacetylate cortactin, thereby promoting autophagosome–lysosome fusion and autophagy (Wang et al., 2019).

There is evidence that reversible acetylation of α-tubulin can regulate microtubule stability and function, which is also essential for the fusion of autophagosomes to lysosomes (Kochl et al., 2006; Xie et al., 2010). In acidic pH–dependent autophagy in cardiomyocytes, decreasing α-tubulin acetylation impairs autophagy maturation and causes cardiomyocyte injury while increasing α-tubulin acetylation can revert the process (Yang et al., 2019). Reactive oxygen species (ROS)–mediated hyperacetylation of microtubule was reported to stimulate autophagy under stress and nutrient starvation (Geeraert et al., 2010; Mackeh et al., 2014). Hyperacetylation of microtubule is mediated by acetyltransferase MEC17 at the residue of lys40 (Mackeh et al., 2014). Spermine increases microtubule acetylation and facilitates selective autophagic degradation of prion aggregates by binding to the microtubule protein Tubb6A (Phadwal et al., 2018). A recent study reported that zinc oxide nanoparticles (a nanomaterial) inhibited autophagy by blocking autophagosome–lysosome fusion, while microtubule acetylation helped promote the autophagic degradation process (Liu J. et al., 2019). Chen et al. (2015) found that potassium bisperoxo (1,10-phenanthroline)oxovanadate [bpV(phen)] suppresses acetylated microtubule–dependent degradation of autophagosomes by disrupting the HDAC6 interacted with p62.

Other researchers have obtained contrasting results. They found that HDAC6-mediated α-tubulin deacetylation was also important for autophagosome maturation. Heterogeneous nuclear ribonucleoprotein K (hnRNPK) deficiency was found to decrease α-tubulin K40 acetylation by HDAC6, consequently enhancing autophagosome–lysosome fusion in the 293-cell line (human renal epithelial cell line) (Li et al., 2018). The ROS pathway has also been reported to reduce the fusion of autophagosome and lysosome by increasing tubulin acetylation (Bonet-Ponce et al., 2016). These findings show that tubulin acetylation does not always enhance autophagy and hyperacetylation of tubulin might impair autophagy flux. The factors leading to the differences in findings between these studies are still not clear. Autophagosomes are usually delivered along the microtubule tracks by dynein to the centrosomes where lysosomes are usually gathered (Geeraert et al., 2010). Since the acetylation during this stage is complex, we speculate that no matter tubulin is acetylated or deacetylated, decreased autophagosome–lysosome formation is due to the disturbed stability of the microtubule system. Thus, balancing the acetylation/deacetylation of tubulin is a key aspect to be considered for autophagy regulation during this phase. Based on these findings, we can conclude that acetylation participates in the entire process of autophagy, from the initial step to the completion of autophagosome degradation.

**FIGURE 1:**
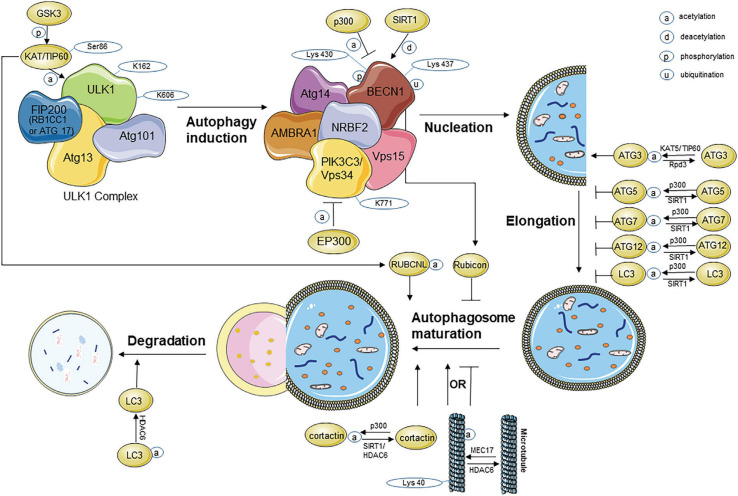
Acetylation modification during the process of autophagy. ATG, autophagy-related protein; BECN1, beclin1; EP300, E1A binding protein p300; FIP200, FAK family kinase-interacting protein of *200* kDa; GSK3β, glycogen synthase kinase-3β; HDAC, histone deacetylase; KAT, lysine acetyltransferase; LC3, microtubule-associated protein light chain 3; PIK3C3, phosphoinositide-3-kinase class 3; RB1CC1, RB1-inducible coiled-coil protein 1; TIP60, Tat-interactive protein 60 kDa; ULK1, unc-51-like kinase 1; VPS, vacuolar protein sorting.

During the early stages, p300/SIRT1 seems to be the major regulator pair, while HDAC6 seems to be the major regulator in the later stages. Therefore, if an p300/SIRT1/HDAC6 inhibitor or activator is used to regulate autophagy, the acetylation condition of the entire process of autophagy will be affected. Because autophagy is a constant process, blocking or activation of any key protein in the process will influence the effective autophagy flux, further indicating that acetylation modification is an important mechanism for regulating autophagy (Figure 1).

### Acetylated FOXO1 and Autophagy Flux

The autophagy process is affected not only by direct acetylation but also by the acetylation of some transcription factors. Fullgrabe et al. (2014) reported several transcription factors related to the transcriptional control of autophagy. Of these, acetylation of transcription factors is best illustrated in the case of forkhead box O (FOXO) family members. FOXO1 is frequently reported to be closely associated with autophagy. Activated FOXO1 enhances autophagy by increasing the expression of numerous autophagy-related genes in various cells (Zhao et al., 2007, 2008; Sengupta et al., 2009; Xu et al., 2011; Warr et al., 2013; Chi et al., 2016; Wang et al., 2018; Liu Y. et al., 2019). Activation of the FOXO family is regulated by complex mechanisms in various cells and tissues, while acetylation is one of them.

SIRT1 directly influences autophagy by deacetylating key components of autophagy-related proteins, including the products of ATG5, ATG7, and ATG8 (Lan et al., 2008). SIRT1 localizes in the nucleus and activates transcription factor from FOXO family, which is also a classic way to induce the expression of autophagy-related proteins (Xiong et al., 2013). Under glucose deprivation conditions, SIRT1 increases FOXO1 deacetylation, thereby increasing the expression of Rab7, which is a small GTP-binding protein that mediates autophagosome–lysosome fusion in the later stages (Hariharan et al., 2010). This is also the mechanism by which resveratrol reverses myocardial oxidative stress injury in diabetic mice (Wang et al., 2014), and by which curcumin protects HUVEC survival from oxidative stress damage (Han J. et al., 2012). The SIRT1/FOXO1/Rab7 axis is also important for maintaining left ventricular function during starvation (Hariharan et al., 2010) and preventing podocyte injury (Majumder and Advani, 2015; Wang et al., 2016) in the same manner. Researchers have found that autophagy induced by cytosolic FOXO1 is capable of suppressing tumor (Zhao et al., 2010) and that, in pancreatic cancer, miR-138-5p inhibits autophagy by blocking the SIRT1/FoxO1/Rab7 axis (Tian et al., 2017). The SIRT1/FOXO1/Rab7 axis comprises a classical pathway that is targeted by researchers to modulate autophagy and to attenuate diseases. Acetylated FOXO1 was found to mediate high glucose induced autophagy in H9C2 cardiomyoblasts as well (Liu et al., 2014).

Another study reported that in QBC939 cells, FOXO1 acetylation and its subsequent interaction with ATG7 regulate basal and serum starvation–induced autophagy, as evidenced by LC3 accumulation and p62 degradation. These findings identify FOXO1 as a potential therapeutic target for treating human cholangiocarcinoma via regulating autophagy (He et al., 2018). In the case of myocardial infarction, total cardiac FOXO1 expression is downregulated at first and partly recovers after 7 days, which is accompanied by fundamental PTMs in FOXO1, particularly acetylation, suggesting that FOXO1 acetylation contributes to cardiac remodeling in post-ischemic heart failure (Kappel et al., 2016). Moreover, Akt2 ablation can protect against cardiac aging through restoring FOXO1-related autophagy (Ren et al., 2017). Liver-specific knockout of glucose-6-phosphatase-α (G6Pase-α; L-G6pc-/-) causes downregulation of SIRT1 signaling and deacetylation of the FOXO family, leading to inactivation of autophagy, which in turn transactivates autophagy genes (Cho et al., 2017). Li J. et al. (2016) found that SIRT3 activation is also essential for reducing the acetylation modification on FOXO1, which in turn alleviates myocardial hypertrophy with chronic angiotensin II (Ang II) infusion through improving autophagy flux.

### Targets for Regulating the Acetylation Modification in Vascular Aging

Vascular aging is one of the main aspects of aging. Endothelial cells (ECs) and vascular smooth muscle cells (VSMCs) are the main structure of blood vessels. The properties of ECs and VSMCs change greatly during vascular aging, gradually leading to compromised vascular function and progressive vascular diseases. Mistriotis and Andreadis (2017) performed an in-depth analysis of the molecular mechanism underlying vascular aging concerning two categories, that is, extrinsic and intrinsic changes. The former category includes chronic inflammation, atherosclerosis, hypertension, vascular wall stiffness, and vascular cell communication. The latter includes telomere attrition, mitochondrial dysfunction, DNA damage, epigenetic changes, nuclear organization loss, and cellular senescence. The role of acetylation in vascular aging, which is the focus of the current review, was not specifically discussed in that study.

From the literature that we reviewed, the existing research on acetylation modification in vascular aging focused on SIRT1. Decreased SIRT1 activity leads to increased lysine acetylation of important targets including p53, endothelial nitric oxide synthase (eNOS), peroxisome proliferator–activated receptor gamma coactivator (PGC)-1α, and matrix metalloproteinase (MMP)-14 at gene or protein level (Cencioni et al., 2015).

### Ameliorating EC Senescence Through Non-autophagy Acetylation Regulation

In H_2_O_2_ induced senescence models of human umbilical vein endothelial cells (HUVECs), 2,3,5,4′-tetrahydroxystilbene-2-O-β-D-glucoside increases blood flow and partly reverse vascular senescence by increasing SIRT1 activity and eNOS expression, thereby decreasing p53 acetylation at the K373 site (Han X. et al., 2012); Ginsenoside Rb1 protects against endothelial senescence and dysfunction through stimulating the expression of SIRT1, which decreases eNOS acetylation and promotes NO production; Furthermore, when SIRT1 is knocked down, the effect of Rb1 on endothelial senescence decreases (Song et al., 2014). On the contrary, elevated trimethylamine-N-oxide (TMAO) levels may induce the EC senescence through decreased SIRT1, which promotes acetylation of eNOS and decreases the level of NO (Ke et al., 2018).

A study using aorta segments isolated from young Wistar rats assessed three different kinds of inhibitors of SIRT1: nicotinamide, sirtinol, and EX527 (Zarzuelo et al., 2013). Inhibitors causes endothelial dysfunction and increases NADPH oxidase–derived ROS in the vascular wall by impairing activities of SIRT1, leading to vascular aging. Moreover, SIRT1 activation decreases PGC-1α acetylation and subsequent PPARα activation, both NADPH oxidase–driven ROS production and NO inactivation decreases, and endothelial function is improved as a result (Zarzuelo et al., 2013). Supplementation with nicotinamide mononucleotide (NMN) in old mice restores endothelium-dependent diastole (EDD) and NO-mediated EDD, reduces brachial-ankle pulse wave velocity (aPWV), normalizes O_2_ production, decreases nitro tyrosine levels, reverses collagen-I deposition, increases elastin levels, and restores vascular SIRT1 activity. And (-)-epicatechin partially restores NO to the levels as in young cells by stimulating SIRT1 binding to the eNOS and decreasing synthase acetylation levels (Ramirez-Sanchez et al., 2018). These findings further prove that SIRT1-mediated deacetylation plays a protective role in vascular aging (de Picciotto et al., 2016).

SIRT1 and liver kinase B1 (LKB1)/AMPK are the two key molecules for regulating the function of EC. In high glucose-induced senescence in mouse microvascular ECs, SIRT1 downregulation plays a crucial role. Reduction of SIRT1 increased FOXO-1 and P53 acetylation, P21 expression. Metformin can protect HG-induced endothelial dysfunction through regulating SIRT1 expression/activity directly or partly via LKB1/AMPK pathway (Arunachalam et al., 2014). What’s more, the protective activities of SIRT1 may also be achieved partly by regulating the acetylation/deacetylation status and stability of LKB1 protein (Zu et al., 2010). In another study, selective overexpression of SIRT1 in the endothelium prevents hypertension and age-related adverse arterial remodeling in mice without eNOS; knockdown of HERC2 abolishes the protective effects by increasing the interaction between LKB1 and transforming growth factor (TGF)-β1 (Bai et al., 2016). From all above, SIRT1 cannot only regulate EC senescence through eNOS/NO pathway but also via LKB1/HERC2 pathway.

### Ameliorating VSMC Senescence Through Non-autophagy Acetylation Regulation

In VSMC cells, Ang II was usually used to induce senescence. Ang II stimulates PGC-1α phosphorylation, leading to the binding of GCN5 to PGC-1α and for its lysine acetylation; Acetylated PGC-1α by Ang II interrupts the PGC-1α-FOXO1-SIRT1 feed-forward signaling circuit, leading to SIRT1 and catalase downregulation and vascular senescence; Thus, endogenous PGC-1α improves vascular hypertrophy by upregulating catalase expression and reducing ROS levels (Xiong et al., 2013). And a new study found that PGC-1α can ameliorate senescence by inducing autophagy in an SQSTM1-dependent manner (Salazar et al., 2020). α7 nicotinic acetylcholine receptor (α7nAChR) alleviates VSMC senescence induced by Ang II by promoting the NAD ^(+)^-SIRT1 pathway, thereby decreasing p53 acetylation (Li D.J. et al., 2016). Neuregulin-1 markedly inhibits H_2_O_2_-induced premature senescence of VSMCs by decreasing p53 acetylation both *in vitro* and in the aorta of mice with diabetes *in vivo* (Shakeri et al., 2018). Exendin-4 increases the acetylation of Nrf2 in a dose-dependent way and the recruitment of the transcriptional coactivator CREBBP to Nrf2; CREBBP silencing attenuates the suppressive effects of exendin-4 on Ang II-induced VSMC senescence and decreases super-oxidant production (Chen et al., 2016). The summarizations are detailed in the following table (Table 1).

**TABLE 1:** The acetylation modification in vascular aging.

**Interventions**	**Senescence induction**	**HAT/HDAC**	**Acetylation substrate**	**Senescence regulation**	**References**
**EC senescence**					
2,3,5,4′-tetrahydroxystilbene-2-O-β-D-glucoside	H_2_O_2_	SIRT1	p53	Ameliorates senescence	Han X. et al., 2012
Ginsenoside Rb1	H_2_O_2_	SIRT1	eNOS	Ameliorates senescence	Song et al., 2014
	TMAO	SIRT1	eNOS	Increases senescence	Ke et al., 2018
SIRT1 inhibitors (nicotinamide, sirtinol, and EX527)	SIRT1 inhibition	SIRT1	PGC-1α	Ameliorates EC dysfunction	Zarzuelo et al., 2013
(-)-epicatechin	Replicative	SIRT1	eNOS	Ameliorates senescence	Ramirez-Sanchez et al., 2018
Metformin	Hyperglycemia	SIRT1	FOXO1&p53	Ameliorates senescence	Arunachalam et al., 2014
	MCM	SIRT3	ATG5	Induces dysfunction	Liu et al., 2018
–	–	HDAC4	FOXO3a	Induces inflammation	Yang et al., 2018
Ginsenoside Rb1	Ox-LDL	SIRT1	BECN1	Ameliorates senescence	Shi et al., 2020
**VSMC senescence**					
–	Ang II	GCN5	PGC-1α	Increases senescence	Xiong et al., 2013
α7nAChR	Ang II	SIRT1	p53	Ameliorates senescence	Li D.J. et al., 2016
Neuregulin-1/Erb4	H_2_O_2_	unknown	P53	Ameliorates senescence	Shakeri et al., 2018
Exendin-4	Ang II	EP300	Nrf2	Ameliorates senescence	Chen et al., 2016
–	–	KAT2A/GCN5	α-Tubulin	Inhibits VSMC migration	Ouyang et al., 2020

*EC, endothelial cell; VSMC, vascular smooth muscle cell; HAT, histone acetylases; HDAC, histone deacetylases; TMAO, Trimethylamine-N-oxide accelerates; Ang II, Angiotensin II; MCM, macrophage-conditioned medium.*

### Targets for Regulating the Acetylation of Autophagy in Vascular Aging

Regarding the acetylation of autophagy in vascular aging, Liu et al. (2018) reported that SIRT3 in macrophages form a molecular complex with ATG5 and acetylation of ATG5 inhibits autophagosome maturation, thus promoting NLRP3 inflammasome activation. The incubation of human aortic ECs with macrophage-conditioned medium induced endothelial dysfunction. These findings revealed that SIRT3-deficient macrophages displayed impaired autophagy and accelerated NLRP3 inflammasome activation and endothelial dysfunction. While another report demonstrated that HDAC4 inhibited vascular inflammation by regulating autophagy in vascular ECs (Yang et al., 2018). This result provides the first evidence that HDAC4 increased rapidly in response to Ang II, and HDAC4 deficiency suppresses activation of autophagy by inhibiting FOXO3a deacetylation, leading to reduced inflammation in Ang II-induced vascular ECs. These evidences indicate SIRT3 or HDAC4 may alleviate vascular aging via regulating acetylation of autophagy. Moreover, Shi et al. (2020) reported Ginsenoside Rb1 alleviated ox-LDL-induced vascular endothelium senescence by regulating SIRT1/BECN1/autophagy, and inhibiting autophagic degradation of KAT2A/GCN5 preventd directional migration of VSMCs (Ouyang et al., 2020). Taken together, regulating the acetylation of autophagy may be a potential way to prevent vascular aging.

## Conclusion and Future Perspectives

Autophagy is a classical pathway involved in many physiological processes, including the elimination of cellular organelles, stimulation of different stress factors, and remodeling of tissues during development. Decreased autophagy creates diverse cellular dysfunctions that exacerbate the aging process, whereas enhanced autophagy generally promotes cellular homeostasis and function to prolong life span and improve health span. Acetylation acts as a novel regulator of autophagy and participates in the whole process of autophagy. Therefore, targeting the acetylation modification during autophagy may be a promising approach for preventing aging and aging-related conditions, such as vascular aging.

The existing research on acetylation modification in vascular aging mainly focuses on SIRT1-mediated non-autophagy–related acetylation of proteins, including acetylation of p53, eNOS, PGC-1α, and Nrf2. Several studies indicate that regulating acetylation of autophagy can also ameliorate vascular aging. Thus, our review aims to provide an integrated view of acetylation modification during the multistep process of autophagy and provide evidence for that regulating autophagy acetylation may be potential therapeutic targets for vascular aging. Finding a way to attenuate vascular aging by modulating autophagy acetylation can provide a bright future (e.g., by helping in increasing their life span, health span, or quality of life) for aging individuals, including prematurely aging people.

## Author Contributions

SZ defined the study theme and methods. JS collected the references and wrote the article. ST, LT, HY, MC, YX, XL, and ZZ were responsible for revising the article and providing valuable suggestions. All authors contributed to the article and approved the submitted version.

## Conflict of Interest

The authors declare that the research was conducted in the absence of any commercial or financial relationships that could be construed as a potential conflict of interest.

## Abbreviations

Ac-CoA: acetyl-CoA
AD: Alzheimer’s disease
AMPK: Adenosine 5′-monophosphate-activated protein kinase
ATG: autophagy-related gene
Ang II: Angiotensin II
α 7nAChR: α 7 nicotinic acetylcholine receptor
aPWV: brachial-ankle pulse wave velocity
BAT3: HLA-B–associated transcript 3
BECN1: beclin1
CMA: chaperone-mediated autophagy
ECs: endothelium cells
eNOS: endothelial nitric oxide synthase
EDD: endothelium-dependent diastole
EP300: E1A binding protein p300
ER: endoplasmic reticulum
FIP200: FAK family kinase-interacting protein of 200 kDa
FOXO: fork head box O
GCN5: general control non-derepressible 5
GSK3β: glycogen synthase kinase-3 β
HATs: histone acetylases
HDACs: histone deacetylases
hnRNPK: heterogeneous nuclear ribonucleoprotein K
HSP: heat shock protein
KAT: lysine acetyltransferase
KAP1: KRAB-ZFP-associated protein 1
LC3: microtubule-associated protein light chain 3
LKB1: liver kinase B1
mTOR: mammalian target of rapamycin
MMP: matrix metalloproteinase
NMN: nicotinamide mononucleotide
NO: nitric oxide
PIK3C3: phosphoinositide-3-kinase class 3
PPARα: peroxisome proliferator-activated receptor α
PGC-1α: peroxisome proliferator-activated receptor gamma coactivator-1 alpha
PTMs: post translational modifications
SIRT: sirtuins
TGF: transforming growth factor
TIP60: Tat-interactive protein 60 kDa
TRIM: tripartite motif
TMAO: trimethylamine-N-oxide
ULK1: unc-51-like kinase 1
VPS: vacuolar protein sorting
VSMCs: vascular smooth muscle cells

## References

[B1] Abdellatif M. Sedej S. Carmona-Gutierrez D. Madeo F. Kroemer G. (2018). Autophagy in Cardiovascular Aging. *Circulat. Res.* 123 803–824. 10.1161/CIRCRESAHA.118.312208 30355077

[B2] Arunachalam G. Samuel S. M. Marei I. Ding H. Triggle C. R. (2014). Metformin modulates hyperglycaemia-induced endothelial senescence and apoptosis through SIRT1. *Br. J. Pharmacol.* 171 523–535. 10.1111/bph.12496 24372553 PMC3904269

[B3] Bai B. Man A. W. Yang K. Guo Y. Xu C. Tse H. F. (2016). Endothelial SIRT1 prevents adverse arterial remodeling by facilitating HERC2-mediated degradation of acetylated LKB1. *Oncotarget* 7 39065–39081. 10.18632/oncotarget.9687 27259994 PMC5129914

[B4] Bánréti A. Sass M. Graba Y. (2013). The emerging role of acetylation in the regulation of autophagy. *Autophagy* 9 819–829. 10.4161/auto.23908 23466676 PMC3672293

[B5] Bonet-Ponce L. Saez-Atienzar S. da Casa C. Sancho-Pelluz J. Barcia J. M. Martinez-Gil N. (2016). Rotenone Induces the Formation of 4-Hydroxynonenal Aggresomes. Role of ROS-Mediated Tubulin Hyperacetylation and Autophagic Flux Disruption. *Mole. Neurobiol.* 53 6194–6208. 10.1007/s12035-015-9509-3 26558631

[B6] Boutouja F. Brinkmeier R. Mastalski T. El Magraoui F. Platta H. W. (2017). Regulation of the Tumor-Suppressor BECLIN 1 by Distinct Ubiquitination Cascades. *Int. J. Mole. Sci.* 18:ijms18122541. 10.3390/ijms18122541 29186924 PMC5751144

[B7] Cencioni C. Spallotta F. Mai A. Martelli F. Farsetti A. Zeiher A. M. (2015). Sirtuin function in aging heart and vessels. *J. Mole. Cell. Cardiol.* 83 55–61. 10.1016/j.yjmcc.2014.12.023 25579854

[B8] Chen M. Zhou X. Yu L. Liu Q. Sheng X. Wang Z. (2016). Low-Level Vagus Nerve Stimulation Attenuates Myocardial Ischemic Reperfusion Injury by Antioxidative Stress and Antiapoptosis Reactions in Canines. *J. Cardiovasc. Electrophysiol.* 27 224–231. 10.1111/jce.12850 26546374

[B9] Chen Q. Yue F. Li W. Zou J. Xu T. Huang C. (2015). Potassium Bisperoxo(1,10-phenanthroline)oxovanadate (bpV(phen)) Induces Apoptosis and Pyroptosis and Disrupts the P62-HDAC6 Protein Interaction to Suppress the Acetylated Microtubule-dependent Degradation of Autophagosomes. *J.Biol. Chem.* 290 26051–26058. 10.1074/jbc.M115.653568 26363065 PMC4646258

[B10] Chen X. Pan Z. Fang Z. Lin W. Wu S. Yang F. (2018). Omega-3 polyunsaturated fatty acid attenuates traumatic brain injury-induced neuronal apoptosis by inducing autophagy through the upregulation of SIRT1-mediated deacetylation of Beclin-1. *J.Neuroinflam.* 15:310. 10.1186/s12974-018-1345-8 30409173 PMC6225685

[B11] Cheng X. Sun Q. (2019). RUBCNL/Pacer and RUBCN/Rubicon in regulation of autolysosome formation and lipid metabolism. *Autophagy* 15 1120–1121. 10.1080/15548627.2019.1596500 30894088 PMC6526810

[B12] Cheng X. Ma X. Zhu Q. Song D. Ding X. Li L. (2019). Pacer Is a Mediator of mTORC1 and GSK3-TIP60 Signaling in Regulation of Autophagosome Maturation and Lipid Metabolism. *Mole. Cell* 73 788.e–802.e. 10.1016/j.molcel.2018.12.017 30704899

[B13] Chi Y. Shi C. Zhao Y. Guo C. (2016). Forkhead box O (FOXO) 3 modulates hypoxia-induced autophagy through AMPK signalling pathway in cardiomyocytes. *Biosci. Rep.* 36:bsr20160091. 10.1042/bsr20160091 27129298 PMC5293586

[B14] Cho J. H. Kim G. Y. Pan C. J. Anduaga J. Choi E. J. Mansfield B. C. (2017). Downregulation of SIRT1 signaling underlies hepatic autophagy impairment in glycogen storage disease type Ia. *PLoS Genet.* 13:e1006819. 10.1371/journal.pgen.1006819 28558013 PMC5469511

[B15] de Picciotto N. E. Gano L. B. Johnson L. C. Martens C. R. Sindler A. L. Mills K. F. (2016). Nicotinamide mononucleotide supplementation reverses vascular dysfunction and oxidative stress with aging in mice. *Aging Cell* 15 522–530. 10.1111/acel.12461 26970090 PMC4854911

[B16] Deribe Y. L. Pawson T. Dikic I. (2010). Post-translational modifications in signal integration. *Nat. Struct. Mol. Biol.* 17 666–672. 10.1038/nsmb.1842 20495563

[B17] Dossou A. S. Basu A. (2019). The Emerging Roles of mTORC1 in Macromanaging Autophagy. *Cancers* 11:cancers11101422. 10.3390/cancers11101422 31554253 PMC6826502

[B18] Esteves A. R. Filipe F. Magalhaes J. D. Silva D. F. Cardoso S. M. (2019). The Role of Beclin-1 Acetylation on Autophagic Flux in Alzheimer’s Disease. *Mole. Neurobiol.* 56 5654–5670. 10.1007/s12035-019-1483-8 30661206

[B19] Fivenson E. M. Lautrup S. Sun N. Scheibye-Knudsen M. Stevnsner T. Nilsen H. (2017). Mitophagy in neurodegeneration and aging. *Neurochem. Int.* 109 202–209. 10.1016/j.neuint.2017.02.007 28235551 PMC5565781

[B20] Fullgrabe J. Klionsky D. J. Joseph B. (2014). The return of the nucleus: transcriptional and epigenetic control of autophagy. *Nat. Rev. Mole. Cell Biol.* 15 65–74. 10.1038/nrm3716 24326622

[B21] Fusco C. Mandriani B. Di Rienzo M. Micale L. Malerba N. Cocciadiferro D. (2018). TRIM50 regulates Beclin 1 proautophagic activity. *Biochim. Biophys. Acta Mole.Cell Res.* 1865 908–919. 10.1016/j.bbamcr.2018.03.011 29604308

[B22] Geeraert C. Ratier A. Pfisterer S. G. Perdiz D. Cantaloube I. Rouault A. (2010). Starvation-induced hyperacetylation of tubulin is required for the stimulation of autophagy by nutrient deprivation. *J. Biol. Chem.* 285 24184–24194. 10.1074/jbc.M109.091553 20484055 PMC2911293

[B23] Han J. Pan X. Y. Xu Y. Xiao Y. An Y. Tie L. (2012). Curcumin induces autophagy to protect vascular endothelial cell survival from oxidative stress damage. *Autophagy* 8 812–825. 10.4161/auto.19471 22622204

[B24] Han X. Ling S. Gan W. Sun L. Duan J. Xu J. W. (2012). 2,3,5,4′-tetrahydroxystilbene-2-O-beta-d-glucoside ameliorates vascular senescence and improves blood flow involving a mechanism of p53 deacetylation. *Atherosclerosis* 225 76–82. 10.1016/j.atherosclerosis.2012.08.011 22981429

[B25] Hariharan N. Maejima Y. Nakae J. Paik J. Depinho R. A. Sadoshima J. (2010). Deacetylation of FoxO by Sirt1 Plays an Essential Role in Mediating Starvation-Induced Autophagy in Cardiac Myocytes. *Circulat. Res.* 107 1470–1482. 10.1161/CIRCRESAHA.110.227371 20947830 PMC3011986

[B26] He W. Zhang A. Qi L. Na C. Jiang R. Fan Z. (2018). FOXO1, a Potential Therapeutic Target, Regulates Autophagic Flux, Oxidative Stress, Mitochondrial Dysfunction, and Apoptosis in Human Cholangiocarcinoma QBC939 Cells. *Cell. Physiol. Biochem.* 45 1506–1514. 10.1159/000487576 29466794

[B27] Hosokawa N. Sasaki T. Iemura S. Natsume T. Hara T. Mizushima N. (2009). Atg101, a novel mammalian autophagy protein interacting with Atg13. *Autophagy* 5 973–979. 10.4161/auto.5.7.9296 19597335

[B28] Huang R. Xu Y. Wan W. Shou X. Qian J. You Z. (2015). Deacetylation of nuclear LC3 drives autophagy initiation under starvation. *Mole. Cell* 57 456–466. 10.1016/j.molcel.2014.12.013 25601754

[B29] Humphrey J. D. Milewicz D. M. (2017). Aging, Smooth Muscle Vitality, and Aortic Integrity. *Circulat. Res.* 120 1849–1851. 10.1161/CIRCRESAHA.117.311075 28596165 PMC5508728

[B30] Iwata A. Riley B. E. Johnston J. A. Kopito R. R. (2005). HDAC6 and microtubules are required for autophagic degradation of aggregated huntingtin. *J. Biol. Chem.* 280 40282–40292. 10.1074/jbc.M508786200 16192271

[B31] Jiang F. (2016). Autophagy in vascular endothelial cells. *Clin. Exp. Pharmacol. Physiol.* 43 1021–1028. 10.1111/1440-1681.12649 27558982

[B32] Jiang Q. Hao R. Wang W. Gao H. Wang C. (2016). SIRT1/Atg5/autophagy are involved in the antiatherosclerosis effects of ursolic acid. *Mole. Cell Biochem.* 420 171–184. 10.1007/s11010-016-2787-x 27514536

[B33] Kappel B. A. Stohr R. De Angelis L. Mavilio M. Menghini R. Federici M. (2016). Posttranslational modulation of FoxO1 contributes to cardiac remodeling in post-ischemic heart failure. *Atherosclerosis* 249 148–156. 10.1016/j.atherosclerosis.2016.04.001 27105158

[B34] Kary C. (2018). Acetylation rules VPS34. *Nat. Cell Biol.* 20:224. 10.1038/s41556-018-0060-0 29476158

[B35] Kawaguchi Y. Kovacs J. J. McLaurin A. Vance J. M. Ito A. Yao T. P. (2003). The deacetylase HDAC6 regulates aggresome formation and cell viability in response to misfolded protein stress. *Cell* 115 727–738. 10.1016/S0092-8674(03)00939-5 14675537

[B36] Ke Y. Li D. Zhao M. Liu C. Liu J. Zeng A. (2018). Gut flora-dependent metabolite Trimethylamine-N-oxide accelerates endothelial cell senescence and vascular aging through oxidative stress. *Free Radical Biol. Med.* 116 88–100. 10.1016/j.freeradbiomed.2018.01.007 29325896

[B37] Khan S. Bhat Z. R. Jena G. (2016). Role of autophagy and histone deacetylases in diabetic nephropathy: Current status and future perspectives. *Genes Dis.* 3 211–219. 10.1016/j.gendis.2016.04.003 30258890 PMC6150107

[B38] Kochl R. Hu X. W. Chan E. Y. Tooze S. A. (2006). Microtubules facilitate autophagosome formation and fusion of autophagosomes with endosomes. *Traffic* 7 129–145. 10.1111/j.1600-0854.2005.00368.x 16420522

[B39] Ktistakis N. T. Tooze S. A. (2016). Digesting the Expanding Mechanisms of Autophagy. *Trends Cell Biol.* 26 624–635. 10.1016/j.tcb.2016.03.006 27050762

[B40] Kuwano K. Araya J. Hara H. Minagawa S. Takasaka N. Ito S. (2016). Cellular senescence and autophagy in the pathogenesis of chronic obstructive pulmonary disease (COPD) and idiopathic pulmonary fibrosis (IPF). *Respir. Investig.* 54 397–406. 10.1016/j.resinv.2016.03.010 27886850

[B41] Lan F. Cacicedo J. M. Ruderman N. Ido Y. (2008). SIRT1 modulation of the acetylation status, cytosolic localization, and activity of LKB1. Possible role in AMP-activated protein kinase activation. *J. Biol. Chem.* 283 27628–27635. 10.1074/jbc.M805711200 18687677 PMC2562073

[B42] Lee J. Y. Koga H. Kawaguchi Y. Tang W. Wong E. Gao Y. S. (2010). HDAC6 controls autophagosome maturation essential for ubiquitin-selective quality-control autophagy. *EMBO J.* 29 969–980. 10.1038/emboj.2009.405 20075865 PMC2837169

[B43] Leidal A. M. Levine B. Debnath J. (2018). Autophagy and the cell biology of age-related disease. *Nat. Cell Biol.* 20 1338–1348. 10.1038/s41556-018-0235-8 30482941

[B44] Li D. J. Huang F. Ni M. Fu H. Zhang L. S. Shen F. M. (2016). alpha7 Nicotinic Acetylcholine Receptor Relieves Angiotensin II-Induced Senescence in Vascular Smooth Muscle Cells by Raising Nicotinamide Adenine Dinucleotide-Dependent SIRT1 Activity. *Arterios. Thromb. Vasc. Biol.* 36 1566–1576. 10.1161/ATVBAHA.116.307157 27339462

[B45] Li J. Chen T. Xiao M. Li N. Wang S. Su H. (2016). Mouse Sirt3 promotes autophagy in AngII-induced myocardial hypertrophy through the deacetylation of FoxO1. *Oncotarget* 7 86648–86659. 10.18632/oncotarget.13429 27880725 PMC5349942

[B46] Li X. Wang Y. Xiong Y. Wu J. Ding H. Chen X. (2016). Galangin Induces Autophagy via Deacetylation of LC3 by SIRT1 in HepG2 Cells. *Sci. Rep.* 6:30496. 10.1038/srep30496 27460655 PMC4962058

[B47] Li Y. Xu W. McBurney M. W. Longo V. D. (2008). SirT1 inhibition reduces IGF-I/IRS-2/Ras/ERK1/2 signaling and protects neurons. *Cell Metab.* 8 38–48. 10.1016/j.cmet.2008.05.004 18590691 PMC2822839

[B48] Li Z. Liu X. Ma J. Zhang T. Gao X. Liu L. (2018). hnRNPK modulates selective quality-control autophagy by downregulating the expression of HDAC6 in 293 cells. *Int. J. Oncol.* 53 2200–2212. 10.3892/ijo.2018.4517 30106132

[B49] Lin S. Y. Li T. Y. Liu Q. Zhang C. Li X. Chen Y. (2012a). GSK3-TIP60-ULK1 signaling pathway links growth factor deprivation to autophagy. *Science* 336 477–481. 10.1126/science.1217032 22539723

[B50] Lin S. Y. Li T. Y. Liu Q. Zhang C. Li X. Chen Y. (2012b). Protein phosphorylation-acetylation cascade connects growth factor deprivation to autophagy. *Autophagy* 8 1385–1386. 10.4161/auto.20959 22717509 PMC3442885

[B51] Liu J. Kang Y. Yin S. Chen A. Wu J. Liang H. (2019). Key Role of Microtubule and Its Acetylation in a Zinc Oxide Nanoparticle-Mediated Lysosome-Autophagy System. *Small* 15:e1901073. 10.1002/smll.201901073 31062916

[B52] Liu J. Tang Y. Feng Z. Hou C. Wang H. Yan J. (2014). Acetylated FoxO1 mediates high-glucose induced autophagy in H9c2 cardiomyoblasts: regulation by a polyphenol-(-)-epigallocatechin-3-gallate. *Metabolism* 63 1314–1323. 10.1016/j.metabol.2014.06.012 25062567

[B53] Liu K. P. Zhou D. Ouyang D. Y. Xu L. H. Wang Y. Wang L. X. (2013). LC3B-II deacetylation by histone deacetylase 6 is involved in serum-starvation-induced autophagic degradation. *Biochem. Biophys. Res. Commun.* 441 970–975. 10.1016/j.bbrc.2013.11.007 24220335

[B54] Liu P. Huang G. Wei T. Gao J. Huang C. Sun M. (2018). Sirtuin 3-induced macrophage autophagy in regulating NLRP3 inflammasome activation. *Biochim. Biophys. Acta Mole. Dis.* 1864 764–777. 10.1016/j.bbadis.2017.12.027 29277324

[B55] Liu X. Klionsky D. J. (2015). TP53INP2/DOR protein chaperones deacetylated nuclear LC3 to the cytoplasm to promote macroautophagy. *Autophagy* 11 1441–1442. 10.1080/15548627.2015.1074373 26213321 PMC4590662

[B56] Liu Y. Li J. Shang Y. Guo Y. Li Z. (2019). CARM1 contributes to skeletal muscle wasting by mediating FoxO3 activity and promoting myofiber autophagy. *Exp. Cell Res.* 374 198–209. 10.1016/j.yexcr.2018.11.024 30500392

[B57] Lopez-Otin C. Blasco M. A. Partridge L. Serrano M. Kroemer G. (2013). The hallmarks of aging. *Cell* 153 1194–1217. 10.1016/j.cell.2013.05.039 23746838 PMC3836174

[B58] Ma Y. Qi M. An Y. Zhang L. Yang R. Doro D. H. (2018). Autophagy controls mesenchymal stem cell properties and senescence during bone aging. *Aging cell* 17:e12709. 10.1111/acel.12709 29210174 PMC5770781

[B59] Mackeh R. Lorin S. Ratier A. Mejdoubi-Charef N. Baillet A. Bruneel A. (2014). Reactive oxygen species, AMP-activated protein kinase, and the transcription cofactor p300 regulate alpha-tubulin acetyltransferase-1 (alphaTAT-1/MEC-17)-dependent microtubule hyperacetylation during cell stress. *J. Biol. Chem.* 289 11816–11828. 10.1074/jbc.M113.507400 24619423 PMC4002089

[B60] Majumder S. Advani A. (2015). The epigenetic regulation of podocyte function in diabetes. *J. Diabet. Complicat.* 29 1337–1344. 10.1016/j.jdiacomp.2015.07.015 26344726

[B61] Matsunaga K. Saitoh T. Tabata K. Omori H. Satoh T. Kurotori N. (2009). Two Beclin 1-binding proteins, Atg14L and Rubicon, reciprocally regulate autophagy at different stages. *Nat. Cell Biol.* 11 385–396. 10.1038/ncb1846 19270696

[B62] Mercer T. J. Gubas A. Tooze S. A. A. (2018). molecular perspective of mammalian autophagosome biogenesis. *J. Biol. Chem.* 293 5386–5395. 10.1074/jbc.R117.810366 29371398 PMC5900756

[B63] Mistriotis P. Andreadis S. T. (2017). Vascular aging: Molecular mechanisms and potential treatments for vascular rejuvenation. *Ageing Res. Rev.* 37 94–116. 10.1016/j.arr.2017.05.006 28579130

[B64] Narita T. Weinert B. T. Choudhary C. (2019). Functions and mechanisms of non-histone protein acetylation. *Nat. Rev. Mole. Cell Biol.* 20 156–174. 10.1038/s41580-018-0081-3 30467427

[B65] Nie T. Yang S. Ma H. Zhang L. Lu F. Tao K. (2016). Regulation of ER stress-induced autophagy by GSK3beta-TIP60-ULK1 pathway. *Cell Death Dis.* 7:e2563. 10.1038/cddis.2016.423 28032867 PMC5260977

[B66] Ouyang C. Mu J. Lu Q. Li J. Zhu H. Wang Q. (2020). Autophagic degradation of KAT2A/GCN5 promotes directional migration of vascular smooth muscle cells by reducing TUBA/α-tubulin acetylation. *Autophagy* 16 1753–1770. 10.1080/15548627.2019.1707488 31878840 PMC8386598

[B67] Pandey U. B. Nie Z. Batlevi Y. McCray B. A. Ritson G. P. Nedelsky N. B. (2007). HDAC6 rescues neurodegeneration and provides an essential link between autophagy and the UPS. *Nature* 447 859–863. 10.1038/nature05853 17568747

[B68] Peserico A. Simone C. (2011). Physical and functional HAT/HDAC interplay regulates protein acetylation balance. *J. Biomed. Biotechnol.* 2011:371832. 10.1155/2011/371832 21151613 PMC2997516

[B69] Phadwal K. Kurian D. Salamat M. K. F. MacRae V. E. Diack A. B. Manson J. C. (2018). Spermine increases acetylation of tubulins and facilitates autophagic degradation of prion aggregates. *Sci. Rep.* 8:10004. 10.1038/s41598-018-28296-y 29968775 PMC6030104

[B70] Pietrocola F. Galluzzi L. Bravo-San Pedro J. M. Madeo F. Kroemer G. (2015). Acetyl coenzyme A: a central metabolite and second messenger. *Cell Metab.* 21 805–821. 10.1016/j.cmet.2015.05.014 26039447

[B71] Plaza-Zabala A. Sierra-Torre V. Sierra A. (2017). Autophagy and Microglia: Novel Partners in Neurodegeneration and Aging. *Int. J. Mole. Sci.* 18:598. 10.3390/ijms18030598 28282924 PMC5372614

[B72] Ramirez-Sanchez I. Mansour C. Navarrete-Yanez V. Ayala-Hernandez M. Guevara G. Castillo C. (2018). (-)-Epicatechin induced reversal of endothelial cell aging and improved vascular function: underlying mechanisms. *Food Funct.* 9 4802–4813. 30129961 10.1039/c8fo00483h PMC6490961

[B73] Ren J. Zhang Y. (2018). Targeting Autophagy in Aging and Aging-Related Cardiovascular Diseases. *Trends Pharmacol. Sci.* 39 1064–1076. 10.1039/C8FO00483H 30458935 PMC6251315

[B74] Ren J. Yang L. Zhu L. Xu X. Ceylan A. F. Guo W. (2017). Akt2 ablation prolongs life span and improves myocardial contractile function with adaptive cardiac remodeling: role of Sirt1-mediated autophagy regulation. *Aging Cell* 16 976–987. 10.1016/j.tips.2018.10.005 28681509 PMC5595687

[B75] Rubinsztein D. C. Marino G. Kroemer G. (2011). Autophagy and aging. *Cell* 146 682–695. 10.1111/acel.12616 21884931

[B76] Sadoul K. Wang J. Diagouraga B. Khochbin S. (2011). The tale of protein lysine acetylation in the cytoplasm. *J. Biomed. Biotechnol.* 2011:970382. 10.1016/j.cell.2011.07.030 21151618 PMC2997609

[B77] Salazar G. Cullen A. Huang J. Zhao Y. Serino A. Hilenski L. (2020). SQSTM1/p62 and PPARGC1A/PGC-1alpha at the interface of autophagy and vascular senescence. *Autophagy* 16 1092–1110. 10.1155/2011/970382 31441382 PMC7469683

[B78] Sebti S. Prebois C. Perez-Gracia E. Bauvy C. Desmots F. Pirot N. (2014). BAT3 modulates p300-dependent acetylation of p53 and autophagy-related protein 7 (ATG7) during autophagy. *Proc. Natl. Acad. Sci. U S A* 111 4115–4120. 10.1080/15548627.2019.1659612 24591579 PMC3964035

[B79] Sengupta A. Molkentin J. D. Yutzey K. E. (2009). FoxO transcription factors promote autophagy in cardiomyocytes. *J. Biol. Chem.* 284 28319–28331. 10.1073/pnas.1313618111 19696026 PMC2788882

[B80] Shakeri H. Gevaert A. B. Schrijvers D. M. De Meyer G. R. Y. De Keulenaer G. W. Guns P. D. F. (2018). Neuregulin-1 attenuates stress-induced vascular senescence. *Cardiovasc. Res.* 114 1041–1051. 10.1074/jbc.M109.024406 29528383

[B81] Shi G. Liu D. Zhou B. Liu Y. Hao B. Yu S. (2020). Ginsenoside Rb1 Alleviates Oxidative Low-Density Lipoprotein-Induced Vascular Endothelium Senescence via the SIRT1/Beclin-1/Autophagy Axis. *J. Cardiovasc. Pharmacol.* 75 155–167. 10.1093/cvr/cvy059 31658172

[B82] Shirakabe A. Ikeda Y. Sciarretta S. Zablocki D. K. Sadoshima J. (2016). Aging and Autophagy in the Heart. *Circulat. Res.* 118 1563–1576. 10.1097/FJC.0000000000000775 27174950 PMC4869999

[B83] Song Z. Liu Y. Hao B. Yu S. Zhang H. Liu D. (2014). Ginsenoside Rb1 prevents H2O2-induced HUVEC senescence by stimulating sirtuin-1 pathway. *PLoS One* 9:e112699. 10.1161/CIRCRESAHA.116.307474 25386949 PMC4227851

[B84] Stern S. Behar S. Gottlieb S. (2003). Cardiology patient pages. Aging and diseases of the heart. *Circulation* 108 e99–e101. 10.1371/journal.pone.0112699 14530186

[B85] Su H. Liu W. (2018). PIK3C3/VPS34 control by acetylation. *Autophagy* 14 1086–1087. 10.1161/01.CIR.0000086898.96021.B9 28980854 PMC6103400

[B86] Su H. Yang F. Wang Q. Shen Q. Huang J. Peng C. (2017). VPS34 Acetylation Controls Its Lipid Kinase Activity and the Initiation of Canonical and Non-canonical Autophagy. *Mole. Cell* 67 907.e–921.e. 10.1080/15548627.2017.1385676 28844862

[B87] Sun T. Li X. Zhang P. Chen W. D. Zhang H. L. Li D. D. (2015). Acetylation of Beclin 1 inhibits autophagosome maturation and promotes tumour growth. *Nat. Commun.* 6:7215. 10.1016/j.molcel.2017.07.024 26008601 PMC4455096

[B88] Sun T. Ming L. Yan Y. Zhang Y. Xue H. (2017). Beclin 1 acetylation impairs the anticancer effect of aspirin in colorectal cancer cells. *Oncotarget* 8 74781–74790. 10.1038/ncomms8215 29088823 PMC5650378

[B89] Tian S. Guo X. Yu C. Sun C. Jiang J. (2017). miR-138-5p suppresses autophagy in pancreatic cancer by targeting SIRT1. *Oncotarget* 8 11071–11082. 10.18632/oncotarget.20367 28052003 PMC5355247

[B90] Wan W. You Z. Xu Y. Zhou L. Guan Z. Peng C. (2017). mTORC1 Phosphorylates Acetyltransferase p300 to Regulate Autophagy and Lipogenesis. *Mole. Cell* 68 323.e–335.e. 10.1016/j.molcel.2017.09.020 29033323

[B91] Wang B. Ding W. Zhang M. Li H. Guo H. Lin L. (2016). Role of FOXO1 in aldosterone-induced autophagy: a compensatory protective mechanism related to podocyte injury. *Oncotarget* 7 45331–45351. 27244896 10.18632/oncotarget.9644 PMC5216726

[B92] Wang B. Yang Q. Sun Y. Y. Xing Y. F. Wang Y. B. Lu X. T. (2014). Resveratrol-enhanced autophagic flux ameliorates myocardial oxidative stress injury in diabetic mice. *J. Cell. Mole. Med.* 18 1599–1611. 10.18632/oncotarget.9644 24889822 PMC4190906

[B93] Wang C. Xu W. Zhang Y. Zhang F. Huang K. (2018). PARP1 promote autophagy in cardiomyocytes via modulating FoxO3a transcription. *Cell Death Dis.* 9:1047. 10.1111/jcmm.12312 30323296 PMC6189197

[B94] Wang R. Tan J. Chen T. Han H. Tian R. Tan Y. (2019). ATP13A2 facilitates HDAC6 recruitment to lysosome to promote autophagosome-lysosome fusion. *J. Cell Biol.* 218 267–284. 10.1038/s41419-018-1108-6 30538141 PMC6314552

[B95] Wani W. Y. Boyer-Guittaut M. Dodson M. Chatham J. Darley-Usmar V. Zhang J. (2015). Regulation of autophagy by protein post-translational modification. *Lab. Investig.* 95 14–25. 10.1083/jcb.201804165 25365205 PMC4454381

[B96] Warr M. R. Binnewies M. Flach J. Reynaud D. Garg T. Malhotra R. (2013). FOXO3A directs a protective autophagy program in haematopoietic stem cells. *Nature* 494 323–327. 10.1038/labinvest.2014.131 23389440 PMC3579002

[B97] Xie R. Nguyen S. McKeehan W. L. Liu L. (2010). Acetylated microtubules are required for fusion of autophagosomes with lysosomes. *BMC Cell Biol.* 11:89. 10.1038/nature11895 21092184 PMC2995476

[B98] Xie Y. Kang R. Sun X. Zhong M. Huang J. Klionsky D. J. (2015). Posttranslational modification of autophagy-related proteins in macroautophagy. *Autophagy* 11 28–45. 10.1186/1471-2121-11-89 25484070 PMC4502723

[B99] Xiong S. Salazar G. Patrushev N. Ma M. Forouzandeh F. Hilenski L. (2013). Peroxisome proliferator-activated receptor gamma coactivator-1alpha is a central negative regulator of vascular senescence. *Arterioscler. Thromb. Vasc. Biol.* 33 988–998. 10.4161/15548627.2014.984267 23430617 PMC3663327

[B100] Xu P. Das M. Reilly J. Davis R. J. J. N. K. (2011). regulates FoxO-dependent autophagy in neurons. *Genes Dev.* 25 310–322. 10.1161/ATVBAHA.112.301019 21325132 PMC3042155

[B101] Yang D. Xiao C. Long F. Su Z. Jia W. Qin M. (2018). HDAC4 regulates vascular inflammation via activation of autophagy. *Cardiovasc. Res.* 114 1016–1028. 10.1101/gad.1984311 29529137

[B102] Yang L. Zhao L. Cui L. Huang Y. Ye J. Zhang Q. (2019). Decreased alpha-tubulin acetylation induced by an acidic environment impairs autophagosome formation and leads to rat cardiomyocyte injury. *J. Mole. Cell. Cardiol.* 127 143–153. 10.1093/cvr/cvy051 30582931

[B103] Yang Y. Fiskus W. Yong B. Atadja P. Takahashi Y. Pandita T. K. (2013). Acetylated hsp70 and KAP1-mediated Vps34 SUMOylation is required for autophagosome creation in autophagy. *Proc. Natl. Acad. Sci.U S A* 110 6841–6846. 10.1016/j.yjmcc.2018.12.011 23569248 PMC3637746

[B104] Yi C. Yu L. (2012). How does acetylation regulate autophagy? *Autophagy* 8 1529–1530. 10.1073/pnas.1217692110 22732483

[B105] Zachari M. Ganley I. G. (2017). The mammalian ULK1 complex and autophagy initiation. *Essays Biochem.* 61 585–596. 10.4161/auto.21156 29233870 PMC5869855

[B106] Zarzuelo M. J. Lopez-Sepulveda R. Sanchez M. Romero M. Gomez-Guzman M. Ungvary Z. (2013). SIRT1 inhibits NADPH oxidase activation and protects endothelial function in the rat aorta: implications for vascular aging. *Biochem. Pharmacol.* 85 1288–1296. 10.1042/EBC20170021 23422569

[B107] Zhang H. Puleston D. J. Simon A. K. (2016). Autophagy and Immune Senescence. *Trends Mole. Med.* 22 671–686. 10.1016/j.bcp.2013.02.015 27395769

[B108] Zhang X. Yuan Z. Zhang Y. Yong S. Salas-Burgos A. Koomen J. (2007). HDAC6 modulates cell motility by altering the acetylation level of cortactin. *Mole. Cell* 27 197–213. 10.1016/j.molmed.2016.06.001 17643370 PMC2684874

[B109] Zhang Y. Zhang M. Dong H. Yong S. Li X. Olashaw N. (2009). Deacetylation of cortactin by SIRT1 promotes cell migration. *Oncogene* 28 445–460. 10.1016/j.molcel.2007.05.033 18850005

[B110] Zhao J. Brault J. J. Schild A. Goldberg A. L. (2008). Coordinate activation of autophagy and the proteasome pathway by FoxO transcription factor. *Autophagy* 4 378–380. 10.1038/onc.2008.388 18227643

[B111] Zhao J. Brault J. J. Schild A. Cao P. Sandri M. Schiaffino S. (2007). FoxO3 coordinately activates protein degradation by the autophagic/lysosomal and proteasomal pathways in atrophying muscle cells. *Cell Metabol.* 6 472–483. 10.4161/auto.5633 18054316

[B112] Zhao Y. Wang L. Yang J. Zhang P. Ma K. Zhou J. (2010). Anti-neoplastic activity of the cytosolic FoxO1 results from autophagic cell death. *Autophagy* 6 988–990. 10.1016/j.cmet.2007.11.004 20798610

[B113] Zhong Y. Wang Q. J. Li X. Yan Y. Backer J. M. Chait B. T. (2009). Distinct regulation of autophagic activity by Atg14L and Rubicon associated with Beclin 1-phosphatidylinositol-3-kinase complex. *Nat. Cell Biol.* 11 468–476. 10.4161/auto.6.7.13289 19270693 PMC2664389

[B114] Zu Y. Liu L. Lee M. Y. Xu C. Liang Y. Man R. Y. (2010). SIRT1 promotes proliferation and prevents senescence through targeting LKB1 in primary porcine aortic endothelial cells. *Circulat. Res.* 106 1384–1393. 10.1038/ncb1854 20203304

